# Pancreatoblastoma: A Descriptive and Comparative Analysis with Pancreatic Ductal Adenocarcinoma Using SEER Data †

**DOI:** 10.3390/cancers18162642

**Published:** 2026-08-16

**Authors:** Abdul Qahar K. Yasinzai, Jordan A. McKean, Grace R. Thompson, Alessandro Paniccia, Austin M. Parrish, Patrick W. Underwood, Gahyun Gim, Steven J. Hughes, Thomas J. George, Ibrahim Nassour

**Affiliations:** 1 Department of Internal Medicine, Louisiana State University Health, Shreveport, LA 71103, USA; 2 University of Florida Health Cancer Institute, Gainesville, FL 32608, USA; patrick.underwood@surgery.ufl.edu (P.W.U.);; 3 Department of Surgery, University of Florida, Gainesville, FL 32608, USAgrace.thompson@surgery.ufl.edu (G.R.T.);; 4 Department of Surgery, University of Pittsburgh Medical Center, Pittsburgh, PA 15213, USA; 5 Department of Medicine, University of Florida, Gainesville, FL 32608, USA

**Keywords:** pancreatoblastoma, pancreatic neoplasms, SEER Program, population-based study, cancer-specific survival, age-stratified outcomes, sex differences in cancer, pancreatic ductal adenocarcinoma, rare pancreatic tumors, pediatric oncology

## Abstract

Background: Pancreatoblastoma (PB) is an exceptionally rare malignant epithelial neoplasm of the pancreas that recapitulates the developing pancreatic anlage. It is defined histologically by acinar-predominant differentiation with characteristic squamoid nests, and it may also show ductal and endocrine differentiation within the same tumor, features that distinguish it from acinar cell carcinoma and solid pseudopapillary neoplasm but that are readily overlooked. It occurs predominantly in young children, although adult-onset disease is well documented. We provide a population-based characterization of PB across the full age spectrum and benchmark it against pancreatic ductal adenocarcinoma (PDAC). Methods: Cases diagnosed between 2000 and 2021 were identified in the Surveillance, Epidemiology, and End Results (SEER) 17-registry database using site and histology codes. Cancer-specific survival (CSS) was estimated and compared, and Cox proportional hazards regression was used to explore associations with cancer-specific mortality. Results: Thirty-nine cases of PB were identified, compared with 155,924 cases of PDAC. The median age at diagnosis was 17 years (range, under 1 to 78 years); 12.8% (n = 5) were younger than 1 year. Males accounted for 69.2% (n = 27) of cases. The cohort was divided at the conventional pediatric-to-adult threshold of 18 years into a pediatric subgroup (age < 18 years; n = 20) and an adult subgroup (age ≥ 18 years; n = 19). CSS at 1 and 5 years was 95.0% and 83.5% in the pediatric subgroup, versus 67.7% and 24.6% in the adult subgroup (log-rank *p* < 0.001). Five-year CSS was 44.8% in males and 75.0% in females. In an exploratory multivariable model, older age was associated with higher cancer-specific mortality both as a dichotomous variable (adjusted hazard ratio [HR] for age ≥ 18 years 10.7, 95% confidence interval [CI] 2.4–48.2; *p* = 0.002) and, in a parallel model, as a continuous variable (adjusted HR 1.4 per 10-year increment, 95% CI 1.1–1.8; *p* = 0.002), indicating an age–mortality gradient. Male sex showed an association in the same direction that did not reach statistical significance (adjusted HR 3.3, 95% CI 0.96–11.6; *p* = 0.06). Relative to PDAC, PB was more frequently diagnosed in males and was associated with markedly superior survival (1- and 5-year CSS 81.8% and 54.9%, versus 28.8% and 4.0%). Conclusions: Pancreatoblastoma is predominantly a malignancy of young males and carries a substantially more favorable prognosis than PDAC, but outcomes differ markedly across the age spectrum, with adult-onset disease showing considerably poorer survival. Translationally, these population-level estimates support age-stratified prognostic counseling, argue for the referral of adults to centers experienced in rare pancreatic tumors, and provide a rationale for prospective molecular profiling to determine whether adult and pediatric PBs are biologically distinct and whether Wnt/beta-catenin pathway activation is therapeutically actionable.

## Simple Summary

Pancreatoblastoma is a very rare cancer of the pancreas that arises from immature pancreatic cells. It is seen most often in young children, but it can also occur in adults. Because so few people are diagnosed each year, almost everything that is known about it comes from case series. Using a large national cancer registry that covers more than one quarter of the United States population, we identified every recorded case diagnosed between 2000 and 2021 and described the patients, their tumors, the treatments they received and how long they survived. We also compared them with patients who had the far more common form of pancreatic cancer, pancreatic ductal carcinoma (PDAC). Survival was much better than for the PDAC but differed markedly between children and adults and between males and females. These population-level estimates may help clinicians counsel families and plan care.

## 1. Introduction

Pancreatoblastoma (PB) is an exceptionally rare malignant neoplasm of the pancreas, accounting for fewer than 0.025% of all pancreatic malignancies or about 0.004 cases per 100,000 people in population-based registries [1]. In absolute terms, this rarity translates to only a small number of incident cases annually in the United States. A recent population-based analysis of the Surveillance, Epidemiology, and End Results (SEER) registries reported an age-adjusted incidence of all pancreatic malignancies in children of 4.24 per 10,000,000 [2]. Pancreatic cancer in general remains one of the most lethal solid-organ malignancies in the United States, with an estimated 66,440 new cases and 51,750 deaths projected in 2024 and a 5-year overall survival rate of only 13%. However, this dismal prognosis is driven almost exclusively by pancreatic ductal adenocarcinoma (PDAC), which constitutes the vast majority of pancreatic malignancies [3]. PB, by contrast, differs from PDAC in virtually every clinicopathological domain. Originally designated “infantile pancreatic carcinoma” in recognition of its predominance in young children, the entity was subsequently renamed pancreatoblastoma to reflect its histological resemblance to the multipotent, predifferentiated pancreatic anlage observed during fetal development [4,5]. Histologically, PB is characterized by acinar-predominant epithelial differentiation punctuated by squamoid nests, the single most distinctive diagnostic feature, with variable ductal and endocrine differentiation and, in a subset of cases, a mesenchymal or heterologous component [6]. Older pathology series estimate that PB constitutes approximately 25% of pancreatic malignancies diagnosed in children [6,7]. Adult PB is reported to exhibit a male predominance in some studies, suggesting potential sex-specific genetic factors contributing to its etiology [8].

The rarity of PB creates diagnostic and therapeutic challenges in routine clinical practice. Histologically, ductal and endocrine differentiation may coexist within the same tumor, reflecting its multipotent cellular origin [6]. Furthermore, congenital PB has been described in association with hereditary overgrowth syndromes, most notably Beckwith–Wiedemann syndrome, a disorder characterized by abnormalities of genomic imprinting at chromosome 11p15 with subsequent dysregulation of growth-regulatory pathways, including IGF2 signaling. At the molecular level, accumulating evidence has also implicated aberrations in the Wnt/beta-catenin signaling axis in PB pathogenesis, with recurrent CTNNB1 mutations and abnormal nuclear beta-catenin accumulation identified in a substantial proportion of tumors [9]. This molecular profile is distinct from that of PDAC. Integrated genomic characterization of PDAC has shown that the dominant recurrent somatic alterations are activating KRAS mutations together with the inactivation of the tumor suppressors TP53, CDKN2A and SMAD4, with CTNNB1 alteration largely confined to the minority of KRAS wild-type tumors [10]. The two entities therefore appear to be driven by different classes of alteration, embryonal developmental and Wnt/beta-catenin pathway dysregulation in PB versus canonical oncogene and tumor-suppressor progression in PDAC. This distinction is not merely descriptive: it underlies the diagnostic value of nuclear beta-catenin immunohistochemistry in separating PB from PDAC and from acinar cell carcinoma, and it explains why the therapeutic strategies developed for PDAC are unlikely to be directly transferable to PB. These observations support the concept that PB may arise through disruptions in embryonal developmental pathways and suggest a distinct molecular biology. Collectively, these findings underscore the importance of further population-level and translational investigations aimed at clarifying the developmental origins, age-related heterogeneity, and biological behavior of this rare malignancy [11,12].

Clinically, PB typically follows an insidious course, with presenting symptoms ranging from nonspecific abdominal pain and weight loss to obstructive jaundice or, less commonly, acute pancreatitis. The specific constellation of signs and symptoms depends on tumor size and anatomic location within the pancreas [8,13]. The head and tail of the pancreas are the most commonly affected sites, contributing to the variability in clinical presentations [14]. Diagnosis typically relies on imaging modalities like ultrasound, computed tomography (CT), or magnetic resonance imaging (MRI), followed by histological confirmation through biopsy or surgical resection [15]. Most patients, particularly adults, present with advanced-stage disease, reinforcing the need for heightened clinical suspicion, early recognition, and prompt multidisciplinary evaluation. Surgical resection remains the cornerstone of curative-intent therapy, with R0 resection associated with the most favorable long-term outcomes, particularly in pediatric patients. Current management of PB is multimodal and, in the absence of any prospective randomized trial in this disease, has been developed largely from accumulated pediatric experience. Complete surgical resection is the only established curative modality. In the setting of unresectable or metastatic disease, platinum- and anthracycline-based chemotherapy regimens have demonstrated clinical activity and are used both to downstage initially unresectable tumors towards resection and in the adjuvant setting; radiotherapy has a limited role, generally reserved for positive surgical margins or palliation, and no standard second-line systemic regimen exists [14,16,17]. Emerging evidence implicates Wnt pathway activation as a potential therapeutic target in adult cases [16,18]. Pediatric patients generally exhibit better survival outcomes, whereas adult patients, particularly males, often face poorer prognoses [17].

The evidence base for PB remains largely restricted to single-institution case reports, small case series, and institutional retrospective cohorts, none of which have the sample size to provide a statistically reliable, population-level characterization of disease demographics, treatment patterns, or prognostic determinants. Using the SEER database, we therefore set out to (i) describe the demographic, clinicopathological and treatment characteristics of PB across the full age spectrum within a single population-based cohort; (ii) quantify cancer-specific survival in younger and older patients with PB and benchmark it against contemporaneously registered PDAC; and (iii) explore, within a deliberately exploratory and hypothesis-generating framework appropriate to the very small number of cases available, which patient characteristics are associated with cancer-specific mortality.

## 2. Methods

### 2.1. Data Source

Data were obtained from the SEER Program database (17 Registries, November 2023 submission, 2000–2021). The 17 SEER registries collectively capture approximately 28% of the U.S. population across geographically and racially diverse regions, enabling population-representative estimates of cancer incidence, treatment, and survival. SEER provides standardized, audited data on tumor histology, stage at diagnosis, treatment receipt, vital status, and cause of death, making it particularly well suited for the study of rare malignancies where prospective data collection is infeasible. The database was accessed on 2 October 2024 under a Data Use Agreement with the National Cancer Institute.

### 2.2. Inclusion Criteria

The International Classification of Diseases for Oncology, Third Edition (ICD-O-3) was used to identify the pancreas as the primary site of malignancy (site code C25.0 through C25.9). Histology code 8971/3 was applied to identify PB cases, and histology codes 8140/3 (adenocarcinoma, NOS) and 8500/3 (infiltrating duct carcinoma) were applied to identify PDAC cases. Only tumors coded as malignant in behavior (behavior code/3) were eligible. No restriction was applied with respect to age, sex, race or ethnicity, stage at diagnosis, or treatment received: PB cases were identified solely by histology code irrespective of age, which is what permits the present cohort to span the full age range from infancy to the eighth decade. All cases meeting these histological and anatomical criteria with available follow-up data were included in the analysis. Histological grade was not used as an inclusion or stratification criterion because grading does not form part of the diagnostic definition of PB and is not routinely assigned to this entity. Because SEER does not perform central pathology re-review, the histological diagnosis is that submitted by the reporting institution. PB and PDAC were analyzed as a combined cohort for comparative analyses and independently for PB age-specific analyses.

### 2.3. Variables

Demographic variables abstracted included age at diagnosis, biological sex, and race/ethnicity. The PB cohort was additionally dichotomized at 18 years, the conventional boundary between childhood and adulthood, into a pediatric subgroup (age < 18 years; n = 20) and an adult subgroup (age ≥ 18 years; n = 19). Tumor characteristics comprised the primary anatomic site within the pancreas, SEER summary stage at diagnosis, and tumor size at resection or biopsy. In accordance with American Joint Committee on Cancer (AJCC) guidance for pancreatic malignancies, a tumor size threshold of 4 cm was used to dichotomize cases into smaller-tumor (≤4 cm) and larger-tumor (>4 cm) subgroups. SEER summary stage, classified as localized, regional (nodal or direct extension), distant (metastatic), or unknown, was used as the staging variable. Treatment-related variables captured whether patients received surgical resection of the primary tumor, systemic chemotherapy, or radiotherapy, as recorded in the SEER database; SEER does not record chemotherapy agent, dose, sequencing relative to surgery, or margin status, and these could not therefore be examined. Survival outcomes included total duration of follow-up in months from diagnosis to last contact, vital status at last follow-up, and cancer-specific survival (CSS) status, defined as death attributable to the index malignancy rather than to competing causes.

### 2.4. Statistical Analysis

All statistical analyses were performed using SAS version 9.4 (SAS Institute Inc., Cary, NC, USA). Continuous variables are reported as medians with the accompanying range and categorical variables as frequencies with percentages. Between-group comparisons of categorical variables were performed using the Pearson chi-square test or Fisher’s exact test, as appropriate based on expected cell frequencies; Fisher’s exact test was applied when any expected cell count was fewer than five. CSS was estimated using SEER*Stat v8.4.5 and is defined throughout as the probability of not dying from the index malignancy, with deaths from other causes treated as censored; the complementary quantity is referred to as cancer-specific mortality. Survival distributions were compared across groups using the two-sided log-rank test. Associations with cancer-specific mortality were examined using Cox proportional hazards regression, reported as hazard ratios (HRs) with 95% confidence intervals (CIs). To ensure that the age–mortality association did not depend on any single cutpoint, age was modeled in two ways: as a dichotomous variable (≥18 vs. <18 years) and, in a parallel model, as a continuous variable (per 10-year increment); sex, ethnicity, and primary tumor site were included as covariates in both. Disease stage was excluded from the multivariable models because of convergence instability arising from quasi-complete separation in the setting of a limited sample size. Surgery and tumor size were likewise not entered: with the number of events available, the addition of further covariates would have further destabilized estimation. Only 18 cancer-related deaths occurred within the PB cohort, which yields fewer than five events per estimated parameter and therefore falls well below the conventional minimum of approximately ten events per variable required for stable maximum-likelihood estimation in Cox regression. The multivariable models are consequently exploratory: their confidence intervals are correspondingly wide, and they are presented as a hypothesis-generating description of the cohort rather than as a source of definitive independent prognostic effects. A two-sided *p*-value less than 0.05 was used as the threshold for statistical significance throughout all analyses. No adjustment was made for multiple comparisons, and given the number of comparisons undertaken in a cohort of this size, the *p*-values should be interpreted descriptively rather than as formal tests of pre-specified hypotheses.

### 2.5. Ethical Considerations

Institutional Review Board (IRB) approval was not required for this study, as the SEER database contains de-identified patient information, ensuring compliance with ethical and confidentiality standards per accessibility agreements.

## 3. Results

### 3.1. Demographic Characteristics and Comparative Analysis Between PB and PDAC

**Figure 1:**
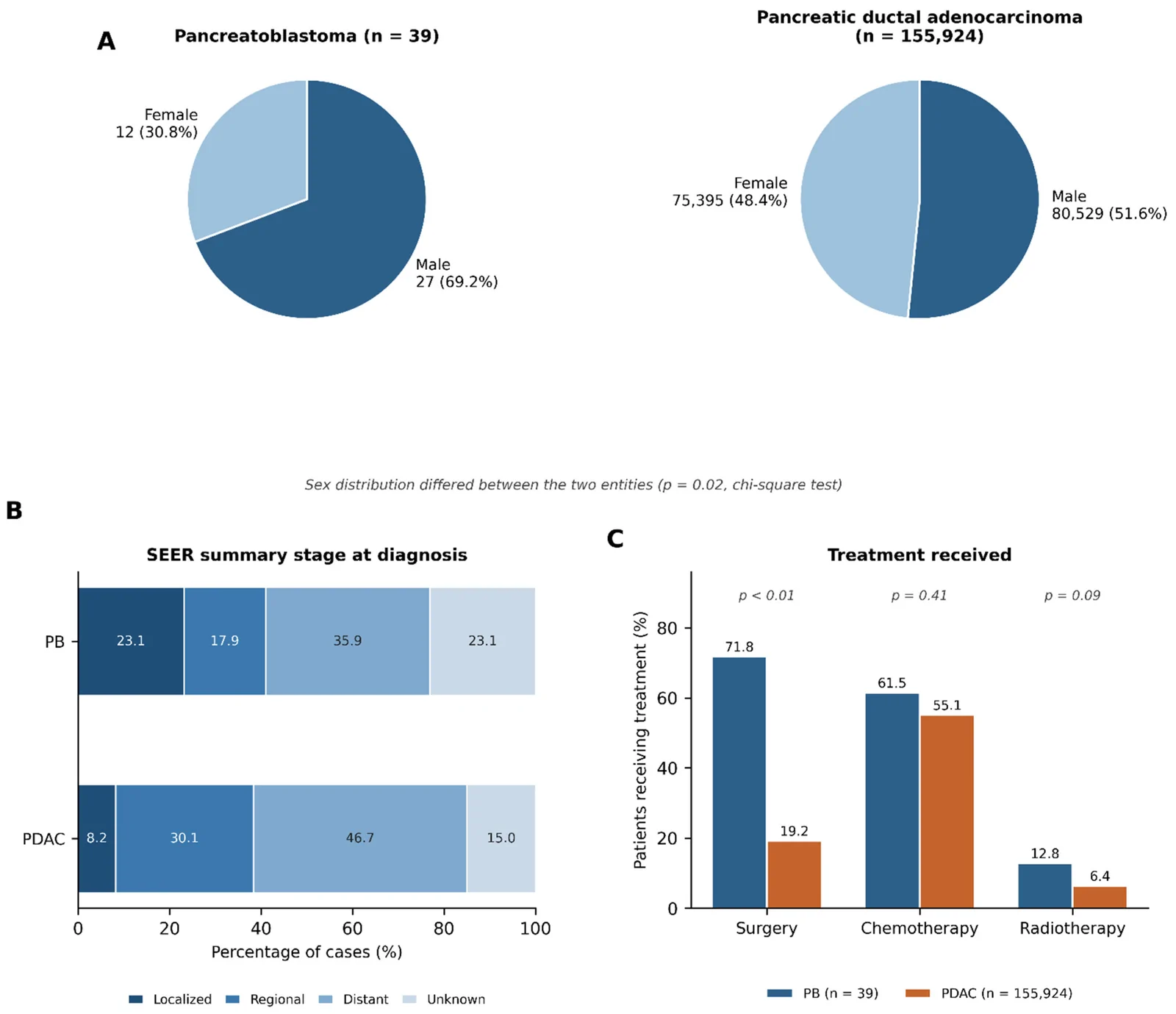
Descriptive comparison of pancreatoblastoma (PB) and pancreatic ductal adenocarcinoma (PDAC). (**A**) Sex distribution in PB and PDAC. (**B**) SEER summary stage at diagnosis. (**C**) Receipt of surgery, chemotherapy and radiotherapy. All values shown are those reported in Table 1. PB, pancreatoblastoma; PDAC, pancreatic ductal adenocarcinoma.

Between 2000 and 2021, the SEER database identified 39 cases of PB, representing just 0.025% of the combined PB and PDAC cohort, compared with 155,924 cases of PDAC. The median age at PB diagnosis was 17 years (0 to 78 years), compared with 70 years (15 to 90 years) for PDAC, with the youngest PB patients diagnosed before the age of 1 year (12.8%; n = 5) and the oldest at 78 years of age (Table 1 and Figure 1).

Sex, race, and ethnic distributions differed between the two entities (Table 1 and Figure 1A). PB was predominantly diagnosed in males (69.2%; *p* = 0.02), with a male-to-female ratio of approximately 2.2:1, whereas PDAC showed a more balanced distribution with a modest male predominance (51.6%). With respect to racial and ethnic distributions, the PB cohort was characterized by a lower proportion of Non-Hispanic White patients (53.8% vs. 69.7%) and a higher representation of Hispanic patients (17.9% vs. 10.9%) compared with the PDAC cohort, respectively. These differences did not reach statistical significance (*p* = 0.35), likely reflecting the limited sample size available for analysis.

### 3.2. Clinical and Pathological Characteristics

PB arose less frequently in the pancreatic head (25.6% vs. 51.8% for PDAC; *p* < 0.01) and more often in the pancreatic tail (23.1% vs. 12.7%; *p* < 0.01). PB cases were more frequently classified as localized disease at diagnosis (23.1% vs. 8.2% for PDAC; *p* < 0.01), whereas distant metastatic disease was less common at diagnosis in PB (35.9% vs. 46.7%; *p* < 0.01), despite a larger tumor size at presentation. PB tumors were significantly more likely to be greater than 4 cm at diagnosis (51.3% vs. 30.4% for PDAC; *p* < 0.01) (Table 1 and Figure 1B).

### 3.3. Treatments

Surgical intervention rates were substantially higher in PB (71.8% vs. 19.2%; *p* < 0.01). Chemotherapy use was similar between the two groups (61.5% vs. 55.1%; *p* = 0.41), while radiotherapy was employed in a minority of PB patients (12.8% vs. 6.4% for PDAC; *p* = 0.09) (Table 1 and Figure 1C).

### 3.4. Pediatric Versus Adult Pancreatoblastoma

The cohort was stratified at 18 years into a pediatric (age < 18 years; n = 20) and an adult (age ≥ 18 years; n = 19) subgroup, as defined in the Methods Section. Pediatric PB patients had a median age of 4 years (range, 0 to 17), reflecting a predominantly early-childhood disease, while adult PB patients had a median age of 51 years (range, 27 to 78), spanning young adulthood to the eighth decade of life. Non-Hispanic White patients predominated among adult cases (73.7%) and were less common among pediatric cases (35.0%), although this difference did not reach statistical significance (*p* = 0.05). No statistically significant differences were identified between pediatric and adult PB in primary tumor site (*p* = 0.36), SEER summary stage (*p* = 0.25), tumor size category (*p* = 0.28), or receipt of surgery (*p* = 0.46), chemotherapy (*p* = 1.00), or radiotherapy (*p* = 0.66) (Table 2).

### 3.5. Survival Analysis

**Figure 2:**
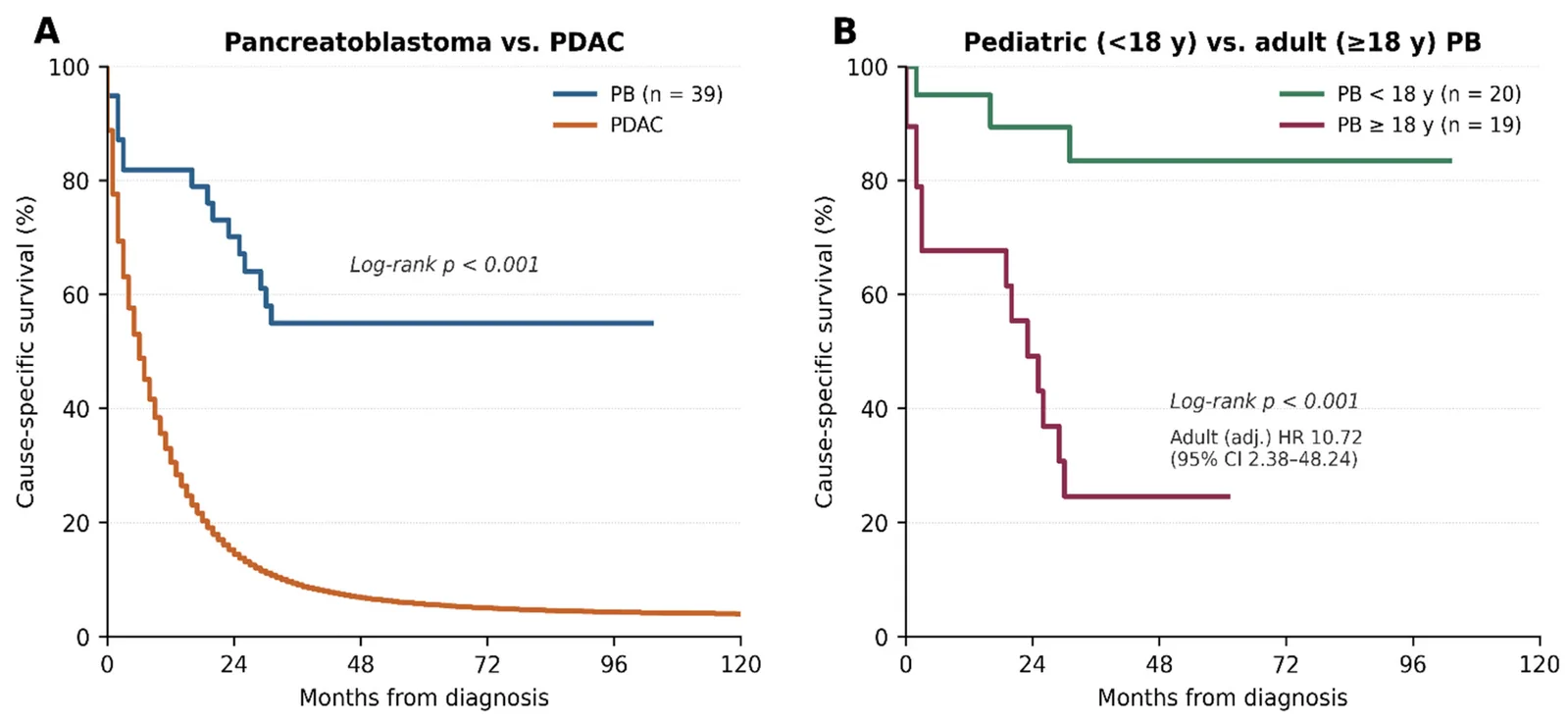
Kaplan–Meier cancer-specific survival analysis of pancreatoblastoma. (**A**) PB versus PDAC. (**B**) Pediatric (<18 years) versus adult (≥18 years) PB. Log-rank *p* < 0.001 for both comparisons; in the adjusted analysis, the hazard ratio for adult age (≥18 years) is 10.7 (95% CI 2.4–48.2). Curves are generated from individual-level SEER data. PB, pancreatoblastoma; PDAC, pancreatic ductal adenocarcinoma.

PB demonstrated a substantial survival advantage over PDAC at all time points. The 1-year CSS was 81.8% for PB versus 28.8% for PDAC; the 2-year CSS was 70.1% versus 12.0%; and the 5-year CSS was 54.9% versus 4.0%, respectively (Table 3 and Figure 2A). Median survival for PB was 19.5 months compared with 5 months for PDAC. Within the PB cohort, pediatric cases exhibited consistently superior survival at every time point examined. The 1-year CSS was 95.0% in pediatric PB versus 67.7% in adult PB, and the 5-year CSS was 83.5% versus 24.6%, respectively (log-rank *p* < 0.001; Figure 2B). Both curves were flat between years 3 and 5, with no further cancer-specific deaths recorded in either subgroup after year 3. Cancer-specific mortality was lower in the pediatric group (20.0%, 4 of 20) than in adults (73.7%, 14 of 19; *p* = 0.001).

In univariable Cox regression, patients aged ≥18 years demonstrated an elevated risk of cancer-specific death compared with those aged <18 years (HR 9.1, 95% CI 2.5–32.9; *p* = 0.001). The corresponding estimate in the multivariable model was 10.7 (95% CI 2.4–48.2; *p* = 0.002). When age was instead modeled as a continuous variable, each additional 10 years of age was associated with a hazard ratio of 1.5 (95% CI 1.2–1.8; *p* < 0.001) in univariable analysis and 1.4 (95% CI 1.1–1.8; *p* = 0.002) after adjustment, indicating that the age–mortality gradient does not depend on the choice of cutpoint. Male sex was associated with worse cancer-specific survival of similar magnitude to the original analysis but did not reach statistical significance (multivariable HR 3.3, 95% CI 0.96–11.6; *p* = 0.06), directionally consistent with the sex-based survival differences observed on Kaplan–Meier analysis. Race and ethnicity and primary tumor site were not associated with survival outcomes in the multivariable model (Table 4). The continuous-age model, whose interval is substantially narrower, provides the more stable characterization, and all estimates should be read as exploratory.

## 4. Discussion

Pancreatoblastoma is a rare and histologically distinct pancreatic malignancy that is not well defined at a population level because of its rarity. Our principal observations, that PB predominates in younger patients, that it carries markedly better survival than PDAC, and that adults fare worse than children, are consistent with both earlier institutional series [2,19]. What the present analysis contributes is the assembly of both children and adults with PB within a single population-based cohort defined by a single histology code and a single registry system, which permits the pediatric and adult subgroups to be compared head to head and both to be benchmarked against PDAC registered over the same period, using cancer-specific rather than overall survival. It also provides a population-level estimate of the sex difference in CSS within PB, which we have not found quantified in the population-based literature.

The multivariable Cox regression identified older age (≥18 years) as the variable most strongly associated with cancer-specific mortality, with a hazard ratio of 10.7 (95% CI 2.4–48.2; *p* = 0.002). With 18 events, several covariates and a confidence interval spanning more than an order of magnitude, the point estimate is poorly determined, and neither its exact value nor the modest change from the univariable estimate of 9.1 should be over-interpreted. Reassuringly, when age was modeled continuously, each additional decade carried an adjusted hazard ratio of 1.4 (95% CI 1.1–1.8; *p* = 0.002), confirming a graded relationship. What the data support, and what is also evident in the unadjusted survival curves, is that older patients in this cohort had substantially worse cancer-specific survival than children (5-year CSS 24.6% vs. 83.5%). Whether this gradient reflects comorbidity and reduced physiological reserve, later diagnosis, or a genuinely more aggressive tumor biology in adult-onset disease cannot be determined from registry data, which contain no information on comorbidity, performance status, symptom duration, mutational landscape, degree of Wnt pathway activation, or immune contexture. We therefore frame these as competing hypotheses requiring prospective molecular and clinical study rather than as an explanation. Similarly, the absence of further cancer-specific deaths beyond year 3 in either subgroup is compatible with a plateau in the hazard, but with so few patients remaining at risk, it cannot be taken as evidence that patients surviving 3 years are cured; the observation is hypothesis-generating and would need to be confirmed with longer follow-up in a larger cohort. Five-year CSS for PB substantially exceeded that of PDAC (54.9% vs. 4.0%), reinforcing the favorable prognostic category that PB occupies relative to the broader pancreatic malignancy landscape when surgical resection is achieved [19]. A male predominance was observed in our cohort (69.2%), and male sex was associated with cancer-specific mortality; however, the association was of borderline significance (multivariable HR 3.3, 95% CI 0.96–11.6; *p* = 0.06), with a corresponding 5-year CSS of 44.8% in males versus 75.0% in females. Male predominance in PB has been variably reported across prior case series [8,19]. Sex differences in cancer incidence and outcome are well documented across many tumor types and are attributable both to circulating sex hormones and to hormone-independent mechanisms, including X-linked gene dosage and escape from X inactivation, sexually dimorphic epigenetic regulation, differences in cellular metabolism, and differences in innate and adaptive immune function [20]. Any of these could in principle underlie the pattern we observed, and the embryonal origin of PB makes developmental and epigenetic mechanisms a particularly reasonable place to look. We emphasize, however, that our data contain no hormonal, genomic or immunological measurements, that the association rests on 18 events, and that we can therefore do no more than identify this as a question worth addressing in a tissue-based study. Our finding that PB tumors were more frequently large (>4 cm) at diagnosis compared with PDAC (51.3% vs. 30.4%; *p* < 0.01) contrasts with the analysis by Yin et al., which did not identify a significant tumor size difference between these entities [19]; the two studies drew on different registries (SEER versus the National Cancer Database) and different age ranges, which may contribute to the discrepancy. Delayed presentation at an advanced disease stage is a plausible contributor to the inferior prognosis of adult PB, a pattern described in the prior single-institution and case series literature [14,16].

PB and PDAC are fundamentally different diseases, and the differences between them are largely predictable. The purpose of our study is not to establish that PB differs biologically from PDAC, which is not in doubt, but to provide a quantitative, contemporaneous, same-registry benchmark against the tumor with which PB is most often conflated in prognostic discussion, so that a clinician confronted with a pancreatic mass in a young patient can attach numbers to the distinction. The comparison shows that PB was managed with curative-intent surgery in nearly three quarters of cases (71.8% vs. 19.2% in PDAC; *p* < 0.01), reflecting younger age, fewer comorbidities, and more frequently resectable tumors. One pattern is genuinely counterintuitive and is not simply a restatement of known differences: PB tumors were more frequently larger than 4 cm at diagnosis (51.3% vs. 30.4%; *p* < 0.01) yet were more often classified as localized (23.1% vs. 8.2%) and less frequently metastatic at presentation (35.9% vs. 46.7%), indicating that tumor size is a poor surrogate for dissemination in this disease. This is consistent with the expansile rather than infiltrative growth habit described for PB, and it carries a practical implication: a large pancreatic mass in a child or young adult should not be assumed to be unresectable. We accept that a comparison of PB with acinar cell carcinoma, the entity with which it shares acinar differentiation and from which it is most difficult to distinguish histologically, would be more biologically informative than the comparison with PDAC, and we regard this as the most valuable next step.

The diagnosis of PB is inherently challenging owing to its rarity, nonspecific clinical presentation, and histological overlap with other solid pancreatic tumors including acinar cell carcinoma and solid pseudopapillary neoplasm. Serum α-fetoprotein (AFP) elevation and hyperlipasemia may provide diagnostically informative clues in selected patients, reflecting the pluripotent differentiation potential of the neoplastic cells [21]. On cross-sectional imaging, PB typically presents as a well-circumscribed, heterogeneous mass with solid and cystic components, central necrosis, and peripheral vascular enhancement on CT; MRI characteristically demonstrates low-to-intermediate T1 signal, high T2 signal, and progressive gadolinium enhancement [22]. These imaging features substantially overlap with those of other solid pancreatic neoplasms, necessitating tissue confirmation for definitive diagnosis. The primacy of surgical resection in PB management is well-established and corroborated by our data, in which over 70% of PB patients underwent operative intervention [8,15]. Achieving complete (R0) resection confers the greatest probability of long-term disease-free survival, particularly in pediatric patients for whom favorable tumor biology augments the surgical benefit. In the setting of locally advanced or metastatic disease, cisplatin- and doxorubicin-based chemotherapy regimens have demonstrated activity in reducing tumor burden and facilitating downstaging to resectability. The limited utilization of radiotherapy in our cohort (12.8%) is consistent with its restricted role in PB management, reserved principally for palliation or adjuvant use in the context of positive surgical margins.

On a molecular level, PB is increasingly recognized as a genomically heterogeneous tumor with recurrent alterations in the Wnt/β-catenin signaling pathway, including CTNNB1 mutations, which may represent actionable therapeutic targets. The well-established association of PB with Beckwith–Wiedemann syndrome, an imprinting disorder arising from dysregulation of the chromosome 11p15.5 region, further supports the role of developmental and epigenetic mechanisms in PB tumorigenesis. The 11p15.5 locus contains several imprinted growth-regulatory genes, most notably IGF2 and CDKN1C, whose altered expression contributes to abnormal cellular proliferation and embryonal tumor predisposition. Although the canonical Wnt/β-catenin pathway genes, including CTNNB1, are not located within the 11p15.5 region (CTNNB1 is located on chromosome 3p22.1), aberrant activation of the Wnt/β-catenin signaling cascade has nevertheless been consistently demonstrated in PB through somatic CTNNB1 mutations and abnormal nuclear β-catenin accumulation [9]. These findings suggest that PB pathogenesis likely reflects cooperative interactions between epigenetic dysregulation of embryonal growth pathways and secondary activation of oncogenic developmental signaling networks. Given these associations, genetic counseling and consideration of germline evaluation may be warranted in patients with congenital or syndromic PB [23]. Case reports have described partial and durable responses to FOLFIRINOX-based regimens in patients with metastatic adult PB harboring Wnt pathway activation, suggesting that genomically informed treatment selection may benefit this population. Emerging targeted therapies and immunotherapeutic strategies represent a promising frontier for PB, and prospective molecular profiling of tumor tissue is warranted [24].

Future perspectives: Three directions follow from these observations. The first is biological: the age and sex differences described here are, at present, purely epidemiological, and establishing whether adult-onset PB is a distinct entity requires paired tumor sequencing and immunophenotyping of pediatric and adult cases, with particular attention to CTNNB1 and the wider Wnt/beta-catenin axis, to 11p15 imprinting, and to the tumor immune microenvironment. The second is infrastructural: because no single center accrues sufficient cases, answering these questions will require an international consortium registry with linked biospecimens and central pathology review, which would simultaneously address the misclassification and missing-covariate problems that limit registry analyses such as ours. The third is clinical: given that PB is chemosensitive and frequently resectable even when large, prospective evaluation of neoadjuvant sequencing in adults, and of genomically directed therapy in Wnt-activated tumors, is warranted, and adults with PB should be considered for referral to centers experienced in rare pancreatic tumors and for enrollment in rare-tumor basket protocols. Until such data exist, the estimates presented here are best used to inform prognostic conversations and to power the design of future studies, not to direct treatment selection.

Several limitations of this study warrant acknowledgment. First, the entire analysis rests on 39 patients and 18 cancer-related deaths. This constrains every estimate we report: subgroup percentages move by 2.6 points with each individual patient, the multivariable model has fewer than five events per parameter, and the resulting hazard ratios carry confidence intervals wide enough that the data are compatible with effects ranging from modest to extreme. The multivariable results should accordingly be read as exploratory and hypothesis-generating and not as establishing independent prognostic effects of age or sex. Second, disease stage, arguably the single most important prognostic variable in any pancreatic malignancy, could not be retained in the multivariable model because of convergence failure, and receipt of surgery and tumor size could not be entered either; the model is therefore not adjusted for the factors most likely to confound the association between age and survival. Third, the retrospective, registry-based design precludes access to granular clinical detail, including specific chemotherapy regimens and their sequencing, surgical margin status, recurrence, and subsequent lines of therapy. Fourth, and directly relevant to the biological questions raised above, SEER records no biomarker or molecular data that includes serum alpha-fetoprotein at diagnosis and during follow-up, CTNNB1 mutation status, nuclear beta-catenin immunohistochemistry, APC status, 11p15 imprinting abnormalities, and any syndromic association such as Beckwith–Wiedemann syndrome or familial adenomatous polyposis; these are all unavailable. No molecular correlation with the demographic and survival patterns we describe is therefore possible within this dataset, and the mechanistic explanations discussed above remain untested. Finally, the SEER 17 registries cover approximately 28% of the United States population, and generalizability beyond that population, and beyond the 2000 to 2021 period over which diagnostic criteria and imaging have changed, should not be assumed. Despite these limitations, this analysis provides population-level estimates for a disease for which the alternative evidence base is single case reports, and the development of prospective, multicenter registries dedicated to rare pancreatic malignancies, potentially through existing national cooperative oncology networks, remains the only realistic route to higher-quality evidence.

## 5. Conclusions

Pancreatoblastoma is a clinically and histologically distinct malignancy that differs from PDAC in its demographics, treatment patterns and prognosis, with a five-year cancer-specific survival of 54.9% compared with 4.0%. Within this small population-based cohort, older age was associated with substantially worse cancer-specific survival, whether age was dichotomized at the conventional 18-year boundary (adjusted HR 10.7, 95% CI 2.4–48.2) or modeled continuously (adjusted HR 1.4 per 10-year increment, 95% CI 1.1–1.8); male sex showed an association in the same direction but did not reach statistical significance. The evidence supports age-aware prognostic counseling and cautions against extrapolating pediatric outcome expectations to adults with this diagnosis. Confirmation in larger collaborative cohorts, together with prospective molecular characterization of the Wnt/beta-catenin pathway and of epigenetic mechanisms, is required before these observations can inform treatment selection.

## Figures and Tables

**Table 1:** Demographic profile and comparative descriptives of pancreatoblastoma and pancreatic ductal adenocarcinoma cases abstracted from the SEER database 2000–2021.

Variables		PB n = 39 (0.025%)	PDAC n = 155,924 (99.97%)	*p*-Value
Median age years (range)		17 (0 to 78)	70 (15 to 90)	-
Gender	Female	12 (30.8%)	75,395 (48.4%)	0.02
	Male	27 (69.2%)	80,529 (51.6%)	
Ethnicity	Non-Hispanic White	21 (53.8%)	108,618 (69.7%)	0.35
	Non-Hispanic Black	6 (15.4%)	17,494 (11.2%)	
	Asian or Pacific Islander	5 (12.8%)	11,700 (7.5%)	
	American Indian or Alaska Native	0 (0%)	883 (0.6%)	
	Hispanic	7 (17.9%)	16,991 (10.9%)	
	Unknown	0 (0%)	238 (0.2%)	
Years of diagnosis	2000–2005	9 (23.1%)	29,359 (18.8%)	0.21
	2006–2010	10 (25.6%)	31,275 (20.1%)	
	2011–2015	4 (10.3%)	38,466 (24.7%)	
	2016–2021	16 (41%)	56,824 (36.4%)	
Cancer-specific death	Death due to this cancer	18 (46.2%)	131,248 (84.2%)	<0.01
	Alive or other causes of death	21 (53.8%)	23,185 (14.9%)	
	Unknown	0 (0%)	1487 (1%)	
Primary site	Head	10 (25.6%)	80,840 (51.8%)	<0.01
	Tail	9 (23.1%)	19,860 (12.7%)	
	Others *	8 (20.5%)	34,793 (22.3%)	
	NOS	12 (30.8%)	20,427 (13.1%)	
SEER summary stage ^x^	Localized	9 (23.1%)	12,754 (8.2%)	<0.01
	Regional	7 (17.9%)	46,971 (30.1%)	
	Distant	14 (35.9%)	72,757 (46.7%)	
	Unknown	9 (23.1%)	23,442 (15%)	
Tumor size	≤4 cm	9 (23.1%)	76,043 (48.8%)	<0.01
	>4 cm	20 (51.3%)	47,335 (30.4%)	
	Unknown	10 (25.6%)	32,546 (20.9%)	
Surgery	Yes	28 (71.8%)	30,000 (19.2%)	<0.01
	No	9 (23.1%)	123,728 (79.4%)	
	Unknown	2 (5.1%)	2196 (1.4%)	
Chemotherapy	Yes	24 (61.5%)	85,925 (55.1%)	0.41
	No	15 (38.5%)	69,999 (44.9%)	
Radiotherapy	Yes	5 (12.8%)	9903 (6.4%)	0.09
	No	34 (87.2%)	145,941 (93.6%)	
Median survival (months)		19.5	5	-

Values are numbers (percentages) unless otherwise stated. * Other sites include the body, overlapping lesions, and ducts of the pancreas. NOS, not otherwise specified. ^x^ SEER summary stage as defined in the SEER manual of 2018; historically, summary stage has also been called general stage, California stage, and historic stage. *p*-values are derived from the chi-square test.

**Table 2:** Demographic profile and comparative descriptives of pediatric and adult pancreatoblastoma cases abstracted from the SEER database 2000–2021.

Variables		PB < 18 Years n = 20 (0.013%)	PB ≥ 18 Years n = 19 (0.012%)	*p*-Value
Median age years (range)		4 (0 to 17)	51 (27 to 78)	-
Gender	Female	6 (30%)	6 (31.6%)	1
	Male	14 (70%)	13 (68.4%)	
Ethnicity	Non-Hispanic White	7 (35%)	14 (73.7%)	0.05
	Non-Hispanic Black	3 (15%)	3 (15.8%)	
	Asian or Pacific Islander	4 (20%)	1 (5.3%)	
	Hispanic	6 (30%)	1 (5.3%)	
Years of diagnosis	2000–2005	4 (20%)	5 (26.3%)	0.05
	2006–2010	8 (40%)	2 (10.5%)	
	2011–2015	0 (0%)	4 (21.1%)	
	2016–2021	8 (40%)	8 (42.1%)	
Cancer-specific death	Death due to this cancer	4 (20%)	14 (73.7%)	<0.01
	Alive or other causes of death	16 (80%)	5 (26.3%)	
Primary site	Head	6 (30%)	4 (21.1%)	0.36
	Tail	3 (15%)	6 (31.6%)	
	Others *	3 (15%)	5 (26.3%)	
	NOS	8 (40%)	4 (21.1%)	
SEER summary stage ^x^	Localized	7 (35%)	2 (10.5%)	0.25
	Regional	2 (10%)	5 (26.3%)	
	Distant	7 (35%)	7 (36.8%)	
	Unknown	4 (20%)	5 (26.3%)	
Tumor size	≤4 cm	3 (15%)	6 (31.6%)	0.28
	>4 cm	10 (50%)	10 (52.6%)	
	Unknown	7 (35%)	3 (15.8%)	
Surgery	Yes	16 (80%)	12 (63.2%)	0.46
	No	3 (15%)	6 (31.6%)	
	Unknown	1 (5%)	1 (5.3%)	
Chemotherapy	No	8 (40%)	7 (36.8%)	1
	Yes	12 (60%)	12 (63.2%)	
Radiotherapy	Yes	2 (10%)	3 (15.8%)	0.66
	No	18 (90%)	16 (84.2%)	
Median survival (months)		23.5	19.5	-

Values are numbers (percentages) unless otherwise stated. * Other sites include the body, overlapping lesions, and ducts of the pancreas. NOS, not otherwise specified. ^x^ SEER summary stage as defined in the SEER manual of 2018; historically, summary stage has also been called general stage, California stage, and historic stage. *p*-values are derived from Fisher’s exact test.

**Table 3:** Five-year cancer-specific survival of pancreatoblastoma and pancreatic ductal adenocarcinoma cases abstracted from the SEER database, 2000–2021.

Cancer-Specific Survival		
Year	PB (n = 39)	PDAC (n = 134,891)
1-year	81.8%	28.8%
2-years	70.1%	12%
3-years	54.9%	6.9%
4-years	54.9%	4.9%
5-years	54.9%	4%
Cancer-Specific Survival		
Year	PB < 18 years (n = 20)	PB ≥ 18 years (n = 19)
1-year	95.0%	67.7%
2-years	89.4%	49.2%
3-years	83.5%	24.6%
4-years	83.5%	24.6%
5-years	83.5%	24.6%

**Table 4:** Cox regression analysis of pancreatoblastoma cases abstracted from the SEER database, 2000–2021. The model is based on 18 cancer-related deaths among 39 patients, and disease stage, receipt of surgery, and tumor size are not included. Age is presented both as a dichotomous variable (≥18 vs. <18 years) and, from a separate model, as a continuous variable (per 10-year increment).

Variables		Univariate Analysis H.R (95% C.I, *p*-Value)	Multivariate Analysis H.R (95% C.I, *p*-Value)
Age	<18 years	Reference	Reference
	≥18 years	9.1 (2.5–32.9, 0.001)	10.7 (2.4–48.2, 0.002)
	Per 10-year increment	1.5 (1.2–1.8, <0.001)	1.4 (1.1–1.8, 0.002)
Gender	Female	Reference	Reference
	Male	2.5 (0.83–7.8, 0.10)	3.3 (0.96–11.6, 0.06)
Ethnicity	Non-Hispanic White	Reference	Reference
	Non-Hispanic Black	0.59 (0.13–2.6, 0.48)	0.37 (0.08–1.8, 0.21)
	Asian or Pacific Islander	0.43 (0.06–3.2, 0.41)	0.69 (0.07–7.2, 0.75)
	Hispanic	0.18 (0.02–1.3, 0.09)	0.31 (0.03–2.8, 0.29)
Primary site	Tail	Reference	Reference
	Head	1.0 (0.36–2.9, 0.96)	1.7 (0.35–7.9, 0.53)
	Others	1.6 (0.51–4.8, 0.44)	0.71 (0.15–3.5, 0.67)
	NOS	0.77 (0.27–2.2, 0.62)	1.2 (0.27–5.0, 0.84)

## Data Availability

This work was accepted for presentation at the AHPBA 2025 annual meeting 2025: A.Q.K. Yasinzai, J.A. McKean, G.R. Thompson, A. Paniccia, A. Parrish, S.J. Hughes, T.J. George, I. Nassour, Pancreatoblastoma of the pancreas: description of a rare clinical entity from the SEER database, HPB, Volume 27, Supplement 1, 2025, Pages S124–S125, ISSN 1365-182X, https://doi.org/10.1016/j.hpb.2025.03.229 (accessed on 23 March 2025).
